# Rhythmic Diel Pattern of Gene Expression in Juvenile Maize Leaf

**DOI:** 10.1371/journal.pone.0023628

**Published:** 2011-08-17

**Authors:** Maciej Jończyk, Alicja Sobkowiak, Paweł Siedlecki, Przemysław Biecek, Joanna Trzcinska-Danielewicz, Jerzy Tiuryn, Jan Fronk, Paweł Sowiński

**Affiliations:** 1 Department of Plant Molecular Ecophysiology, Institute of Plant Experimental Biology and Biotechnology, Faculty of Biology, University of Warsaw, Warsaw, Poland; 2 Plant Biochemistry and Physiology Department, Plant Breeding and Acclimatization Institute - National Research Institute, Radzików, Poland; 3 Department of Plant Molecular Biology, Institute of Plant Experimental Biology and Biotechnology, Faculty of Biology, University of Warsaw, Warsaw, Poland; 4 Institute of Applied Mathematics and Mechanics, Faculty of Mathematics, Informatics, and Mechanics, University of Warsaw, Warsaw, Poland; 5 Department of Molecular Biology, Institute of Biochemistry, Faculty of Biology, University of Warsaw, Warsaw, Poland; 6 Institute of Informatics, Faculty of Mathematics, Informatics, and Mechanics, University of Warsaw, Warsaw, Poland; Instituto de Biología Molecular y Celular de Plantas, Spain

## Abstract

**Background:**

Numerous biochemical and physiological parameters of living organisms follow a circadian rhythm. Although such rhythmic behavior is particularly pronounced in plants, which are strictly dependent on the daily photoperiod, data on the molecular aspects of the diurnal cycle in plants is scarce and mostly concerns the model species *Arabidopsis thaliana*. Here we studied the leaf transcriptome in seedlings of maize, an important C4 crop only distantly related to *A. thaliana*, throughout a cycle of 10 h darkness and 14 h light to look for rhythmic patterns of gene expression.

**Results:**

Using DNA microarrays comprising *ca.* 43,000 maize-specific probes we found that *ca.* 12% of all genes showed clear-cut diel rhythms of expression. Cluster analysis identified 35 groups containing from four to *ca.* 1,000 genes, each comprising genes of similar expression patterns. Perhaps unexpectedly, the most pronounced and most common (concerning the highest number of genes) expression maxima were observed towards and during the dark phase. Using Gene Ontology classification several meaningful functional associations were found among genes showing similar diel expression patterns, including massive induction of expression of genes related to gene expression, translation, protein modification and folding at dusk and night. Additionally, we found a clear-cut tendency among genes belonging to individual clusters to share defined transcription factor-binding sequences.

**Conclusions:**

Co-expressed genes belonging to individual clusters are likely to be regulated by common mechanisms. The nocturnal phase of the diurnal cycle involves gross induction of fundamental biochemical processes and should be studied more thoroughly than was appreciated in most earlier physiological studies. Although some general mechanisms responsible for the diel regulation of gene expression might be shared among plants, details of the diurnal regulation of gene expression seem to differ between taxa.

## Introduction

The Earth environment undergoes periodic changes, such as diurnal, lunar and solar cycles. Living organisms have not only adapted to these changes but also developed mechanisms to sense the cyclic signals from the environment allowing proper adjustment of their metabolism, growth and development. These mechanisms involve endogenous oscillators and other clocks. These oscillators are self-sustaining, but can also be synchronized by external stimuli, usually light and temperature. Light is particularly important for plants, since it is not only a source of information on the state of the environment, but also the source of energy for these photoautotrophic organisms.

Biological rhythms in plants manifest mostly as seasonal and circadian rhythms [1]. The former depend chiefly on light receptors of blue and red/far red light and participate in the regulation of yearly metabolic shifts, *e.g.*, onset of dormancy, and developmental processes, *e.g.*, flowering. Circadian rhythms participate in diurnal regulation of metabolism [2], [3] and have been studied mainly in *Arabidopsis thaliana*, a model plant [4]–[9]. The molecular data gathered so far concerns identification of circadian oscillators as well as diurnal transcriptome changes [10], [11]. *Brassicaceae*, the dicot family to which *A. thaliana* belongs, are evolutionarily distant from the monocot family *Poaceae*. The latter is of utmost interest since it groups major contemporary crop species. The monocots and dicots diverged *ca.* 130 my ago [12] and differ fundamentally in a number of anatomic and physiological features. Additionally, in respect to photoperiodism, *A. thaliana* is a long-day plant, while maize, being of tropical origin, is a short-day plant. This suggests that there could be substantial differences in the regulatory mechanisms underlying their diurnal rhythms. On the other hand, some basic biological mechanisms are remarkably conservative and in fact are shared by organisms much more distantly related than are monocots and dicots. One clearly needs more comprehensive data for monocots to compare them with dicots (see, *e.g.*, discussion in [13], [14]). Until recently, the rather fragmentary information regarding circadian oscillations in plants indicated both similarities and differences between grasses and *A. thaliana* [15]. Even less was known on the global diel patterns of gene expression in monocots.

Recent years have seen a rapid accumulation of studies addressing the problem of circadian regulation of gene expression in plants other than *A. thaliana* and also first attempts at comparative analyses. A comprehensive DIURNAL project comprising specialized analytical tools was initiated in 2007 [16] to study diurnal gene expression patterns in different plants and search for conserved mechanisms. A comparative study of diel transcriptome changes in rice, poplar and *A. thaliana* using the DIURNAL tools has been published lately showing that, in addition to universal mechanisms, some species-specific diversification of diurnal/circadian-associated transcriptional circuits may exist [17].

Last year saw a simultaneous publication of two independent papers concerning diel changes of the maize transcriptome, using different experimental designs and plants of substantially different developmental stages [18], [19]. One study [19] investigated whole shoots of one-week-old seedlings in constant light and temperature, the second [18] was conducted in the field and analyzed adult leaf and developing ears. Both papers stressed conservation of circadian clock mechanisms between maize and *A. thaliana*.

Here, we studied diel transcriptome changes in a defined part of the third (first fully autotrophic, [20]) juvenile leaf of maize grown in controlled conditions under a close-to-physiological setup of 14 h light/10 h dark and 24°C/22°C temperature. We used microarrays representing a major portion of the maize transcriptome and the microarray data were analyzed to arrive at biologically-oriented conclusions. Latest data from the Maize Genome Sequencing Project [21] were used for annotation of transcripts.

Data cluster analysis was performed to define groups of transcripts with similar expression time-profiles. A bioinformatic analysis of potential regulatory regions of those genes was done to search for underlying molecular regulatory mechanisms. Functional analysis was performed with the hierarchical Gene Ontology classification to identify functionally relevant over-represented groups of genes among the clusters identified. Individual genes showing diurnal cycling were additionally characterized using the Ariadne Pathway Studio program.

## Materials and Methods

### Plant material

Dent type CM 109 inbred line of maize (*Zea mays indentata*) was used. This line had been used before in physiological and molecular studies ([22] and citations there). Kernels were germinated in wet sand in darkness at 25°C. Seedlings were transferred to pots containing Knop's nutrient solution supplemented with Hoagland's micro-nutrients. Further growth was conducted in a growth chamber (photoperiod: 14 h/10 h, light irradiance: 250 µmol quanta·m^−2^·s^−1^, day/night temperature: 24°C/22°C). After full development of the third leaf (fully developed ligular region) the plants were used for experiments. The experiment was begun at the start of the dark period (time zero), at which time a large sample (eight plants) was taken to serve as a reference in hybridizations. Further samples were taken after 200, 400, 600, 810, 1,020, 1,230 and 1,440 minutes of growth (total 24 hours). Each sample consisted of the middle part of the third leaf blade, pooled from three plants and frozen in liquid nitrogen. Four fully independent consecutive (late spring to early autumn) experiments were performed, beginning from sowing through plant cultivation, sample preparation to microarray hybridization.

### Microarray description

We used two-color oligonucleotide microarrays designed and produced by the Maize Oligonucleotide Array Project (University of Arizona, Tucson, USA, www.maizearray.org; [23]), comprising 46,128 mainly 70-mer and some 40- and 50-mer probes, and also positive, negative and print controls printed on a glass slide. The probes represented maize cDNAs, ESTs, genes and gene fragments, generally in a one-to-one relation. In some cases, however, two or even more probes corresponded to a single transcript (see Table S1).

### RNA preparation and hybridization

RNA was isolated and purified from frozen leaf samples with RNeasy Plant Mini Kit (Qiagen) following manufacturer's manual. RNA isolation, amplification, labeling and hybridization to microarrays followed the procedure posted on the Maize Microarray Project website (www.microarray.org) with minor modifications [22]. The hybridizations were done in reference design. Each of the seven time-point samples was hybridized with the reference time zero sample. To cancel the effect of potential dye-bias, labeling was done with dye swap: two labelings of reference-cy3 *vs* sample-cy5 and two opposite ones. In all, 28 hybridizations were done: a series of seven for each of the four independent biological experiments. Slide scanning was done with a GenePix 4000B scanner and feature extraction was done with the GenePix Pro 3.0 software (Axon Instruments).

### Data normalization and identification of differentially expressed genes

To allow meaningful comparison and averaging of results obtained on individual microarrays, a two-step normalization was performed. Values of spot fluorescence with local background fluorescence subtracted were imported to Acuity 4.0 (Axon Instruments) and loess-normalized within slides (print-tip loess). The within-slide normalized data was exported to JMP Genomics 6.0.3 (SAS Institute) and loess-normalized between slides. Transcripts whose expression changed in a statistically significant manner in subsequent time points were identified by means of mixed model ANOVA followed by multiple comparisons with false discovery rate correction [24] set at 0.01 or 0.05 (JMP Genomics 6.0.3). Transcript levels at each time point were compared with the level in the time-zero sample. Microarray experiments were described in compliance to MIAME (Minimum Information About Microarray Experiment; [25]) guidelines. Raw microarray data have been deposited at the ArrayExpress database (www.ebi.ac.uk/arrayexpress) under experiment accession number: E-MEXP-3212 and array design number: A-MEXP-2054.

### Clustering

Clustering was conducted with the agglomerative hierarchical clustering method [26] with the complete linkage setup and dissimilarity matrix derived from pairwise correlations. Different numbers of clusters and different dissimilarity measures were examined. The final clustering was chosen with the support of the pvclust method, but without any strict threshold for p-values. The pvclust method makes use of the multiscale bootstrap technique to verify the stability of chosen clustering (R project with MASS, cluster and pvclust packages [27], [28]). With this approach the number of clusters is optimized so that they are the most informative, striking a balance between their number and homogeneity.

### Promoter regulatory element sequence motif analysis

The microarray probe sequences were fetched from the Operon website (omad.operon.com/download/index.php) and the corresponding gene sequences were downloaded from www.maizesequence.org. The probes were matched with the genes using the BLAST software suite. The resulting matches were divided into three distinct groups: a) probes that match a single gene – a gene group called “single”, b) probes matching more than one gene – “multiple”, and c) probes that do not match any known or predicted gene, or match with a poor e-value (>1e-20) – the “none” group. Promoter sequences (defined as 500 bp upstream of first ATG) were fetched from www.maizesequence.org for a search for potential regulatory elements. A program was written in PERL to automate the whole workflow described above (available from the authors upon request).

To discover likely transcription factor-binding sites comparative genomics was used. The maize promoters were aligned with the corresponding sequences of orthologous genes from *Sorghum bicolor*. Data from www.maizesequence.org was used to define *S. bicolor* orthologs and fetch corresponding promoter sequences. The paired promoter sequences from *Z. mays* and *S. bicolor* were exported to ConSite [29] which searches for potential transcription factor-binding sites shared by the two sequences in high homology areas. Only sequences 100% identical with experimentally defined consensus sequences for a known transcription factor, or sequences with a single miss-match with a consensus, were considered. This conservative approach was chosen to avoid short sequence-motifs conserved by chance and corresponding to transcription factor-binding sites of unknown specificity. The exact Fisher test was used to calculate p-values for enrichment of a given transcription factor binding sequence obtained from ConSite in maize genes grouped in each cluster (see above). The null hypothesis was that a given transcription factor binding sequence occurred in a given cluster with the same probability as for all other clusters.

Additionally to the above analysis, sequences 500 bp upstream of the first ATG from all maize genes were searched (on both DNA strands and in both directions) for CBS (CCA1 binding site, AA(A/C)AATCT) and EE (evening element, AAAATATCT), shown previously to drive rhythmic gene expression in *A. thaliana* [4], [30].

### Gene Ontology annotation and enrichment analysis

The oligos (probes) represented on the microarrays were annotated using the latest data from the Maize Genome Sequencing Project (www.maizesequence.org; version 5a.59). The oligos (omad.operon.com/download/index.php) were matched (formatdb and blastn programs) to cDNA sequences from the Maize Genome Sequencing Project. Results were filtered to include only probes whose alignment to cDNA fulfilled all of the following criteria: (1) e-value<0.001; (2) no gaps in alignment; (3) identity ≥50%; (4) alignment length ≥50%. For each oligo the best alignment satisfying the above criteria was chosen. If for a given oligo there were multiple full (*i.e.*, 100%) alignments, all were retained. Ninety-six percent of all oligos satisfied the filtering conditions and were then matched with the corresponding Gene Ontology annotations available on the Maize Genome Sequencing Project website. As a result, 57% of all oligos obtained GO annotations. Over-represented (enriched) GO categories were detected with the Ontologizer (compbio.charite.de/index.php/ontologizer2.html; [31]) program using the “Parent-Child Union” method which takes into account the hierarchical structure of the GO system, and false discovery rate correction set at 0.05 [24]. Only non-redundant sequences were considered, *i.e.*, when several oligos matched a single cDNA, it was counted only once. The GO graphs were drawn with the GraphViz program (www.graphviz.org).

### Pathway analysis

Pathway analysis was done with Pathway Studio 8.0 (Ariadne Genomics). To use this program the identifiers of the genes of interest had to be converted into sequence ids recognizable by the software. First, the microarray oligos were matched with the data from the Maize Genome Sequencing Project as described above. From this database we retrieved identifiers (UniProt, RefSeq Peptide) and mapped them to the ids on the UniProt website (EntrezGene, GenBank, KEGG, Unigene). With this approach *ca.* 70% of the oligos represented on the microarrays used in this study could be subjected to further pathway analysis of gene interactions and interactions with known transcription factors and miRNA.

## Results

The juvenile maize leaf transcriptome demonstrated strong diurnal rhythmicality. The microarray analysis showed that, depending on the level of statistical significance assumed, 5,154 (FDR = 0.01) or 10,514 (FDR = 0.05) of the 43,393 probes (∼12% and ∼24%, respectively) indicated a changed expression of their cognate transcripts during the 24-h period. In all further considerations we use the more stringent criteria of statistical significance (FDR = 0.01).

### Cluster analysis

**Figure 1:**
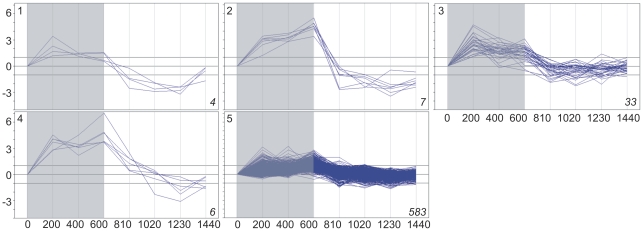
Clustering of diel time-profiles of gene expression in maize leaves in cluster group “night” (clusters 1–5). Ratio of expression level at a given time to the reference level at time 0 is shown as log_2_[sample/reference]. Dark period 0–600 min, light period 600–1440 min. Number of profiles in a given cluster is shown in *italics* in right-hand bottom corner of each plot.

**Figure 2:**
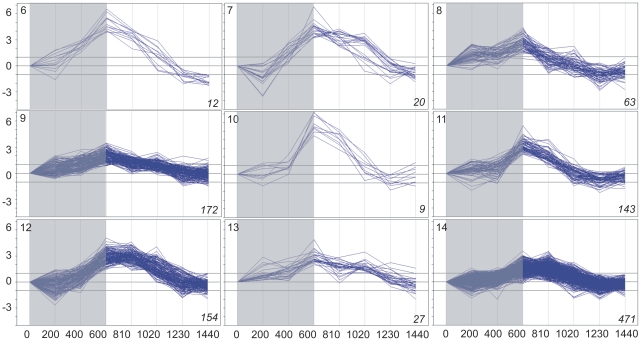
Clustering of diel time-profiles of gene expression in maize leaves in cluster group “dawn” (clusters 6–14). Ratio of expression level at a given time to the reference level at time 0 is shown as log_2_[sample/reference]. Dark period 0–600 min, light period 600–1440 min. Number of profiles in a given cluster is shown in *italics* in right-hand bottom corner of each plot.

**Figure 3:**
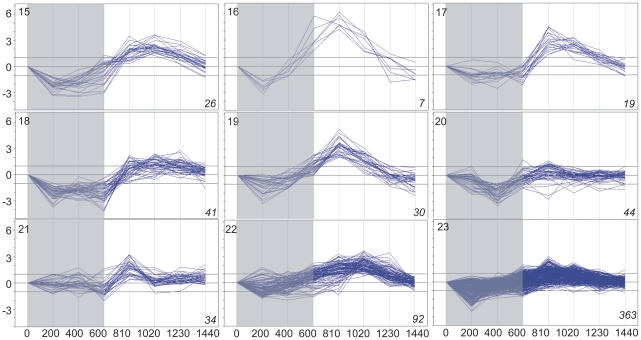
Clustering of diel time-profiles of gene expression in maize leaves in cluster group “day” (clusters 15–23). Ratio of expression level at a given time to the reference level at time 0 is shown as log_2_[sample/reference]. Dark period 0–600 min, light period 600–1440 min. Number of profiles in a given cluster is shown in *italics* in right-hand bottom corner of each plot.

**Figure 4:**
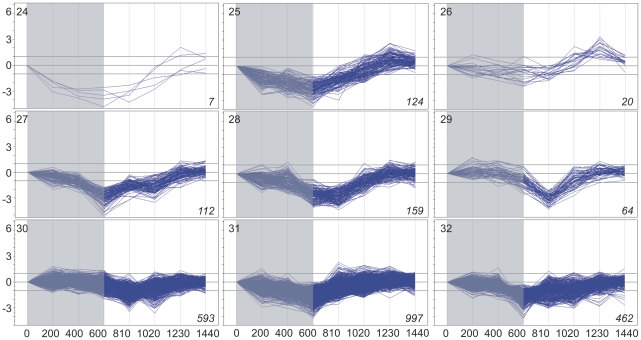
Clustering of diel time-profiles of gene expression in maize leaves in cluster group “dusk” (clusters 24–32). Ratio of expression level at a given time to the reference level at time 0 is shown as log_2_[sample/reference]. Dark period 0–600 min, light period 600–1440 min. Number of profiles in a given cluster is shown in *italics* in right-hand bottom corner of each plot.

Basing on the above data on gene expression, the subset of 5,154 genes whose expression changed significantly at least once during the 24 h was selected. Cluster analysis divided this subset into 35 clusters, each grouping genes with expression time-profiles of similar shape and amplitude (fold difference between the maximal and minimal level of a given transcript within the 24-h period). Three such clusters comprised 258 genes with low-amplitude profiles of expression and for this reason have been discarded from further analysis. The remaining 32 clusters (4,898 genes altogether; Table S1 lists all these genes), each comprising between four and 997 genes, are illustrated in Figures 1, 2, 3 and 4. For convenience, they will be further referred to by their numbers in **bold** typeface. The amplitude of transcript levels was very high for some clusters, the highest value being close to 100 [log_2_(maximum)−log_2_(minimum)>6]. Genes with high amplitudes of expression (>16) were generally those showing expression peak during the dark period (clusters **2**,**4**,**6**,**7**,**10**), *i.e.*, during the first 10 h (600 min) of experiment.

The changes in expression of the genes chosen for analysis were clearly of a cyclic character, as the level of almost all transcripts returned to the initial (the reference at time zero) values at the end of the 24-h period. With the exception of cluster **21**, all genes showed rather gradual accumulation and then depletion of their transcripts, often over ten or more hours, but always with a clear-cut maximum. Basing on the time-point of the maximal transcript accumulation, four groups of clusters were easily discernible: those peaking throughout the dark phase of photoperiod, at the end of the dark phase, during the first half of the light phase, and during the second half of the light phase. For convenience, those groups will be further referred to as, respectively, night, dawn, day, and dusk. The night group comprised clusters **1–5** (633 genes), dawn – clusters **6–14** (1,071 genes), day – clusters **15–23** (656 genes), and dusk – clusters **24–32** (2,538 genes). Figures 1, 2, 3 and 4 show the 32 clusters manually assembled into these four groups. This grouping highlighted a non-uniform distribution of the temporal expression patterns of the genes and also enabled further statistically sound Gene Ontology analyses. The most common (concerning the highest number of genes), and also the most pronounced (high-amplitude) expression maxima were observed towards and during the dark phase: 51% of the cycling genes peaked at dusk and 13% around midnight. A substantial group of genes peaked at dawn (∼22%). The fraction of genes showing maximum of expression during the day was only *ca.* 14% of the entire set of the cycling ones.

### Detailed and global analysis of selected gene groups

All four cluster groups (night, dawn, day, and dusk) were analyzed in respect to the functions of the genes they group. To do that, we performed a global analysis looking for over-represented (enriched) Gene Ontology categories in each cluster group. Results of the GO analysis are shown in Figures S1, S2, S3, S4, S5, S6 and S7. Individual genes from the over-represented GO categories are listed in supplementary material (Table S2).

Among the 633 night-peaking genes the vast majority (cluster **5**, 583 genes) showed a low amplitude of expression (*ca.* 2). A small portion (50 genes, clusters **1,2,3,4**), however, showed high (8–32 times) amplitude of expression. Twenty-five of the highly changeable genes had an Entrez Gene ID; among them four encoded transcription factors and three were signal transduction-related. Global analysis of all the genes in the night cluster group found only one over-represented lowest-rank GO category (only the lowest-rank GO categories are discussed, as they are the most informative), GO:0006464 (protein modification process) from the “biological process” GO class (Figure S1).

Dawn-peaking genes (1071) were grouped in nine clusters of very high (up to 64, clusters **6,7,10**) or moderate (4–16, clusters **8,9,11,12,13,14**) amplitude of expression. Although all genes in this cluster group peaked at dawn, expression of genes grouped in three clusters (**7,8,9**) began to increase already around midnight. All the genes in the dawn cluster group remained induced until midday. In this cluster group several lowest-rank GO categories were over-represented (Figures S2, S3): GO:0006021 (inositol biosynthetic process), GO:0006464 (protein modification process) and GO:0006839 (mitochondrial transport) from the “biological process” GO class, and GO:0016872 (intramolecular lyase activity), GO:0042578 (phosphoric ester hydrolase activity), GO:0015200 (methylammonium transmembrane transporter activity), GO:0051739 (ammonia transmembrane transporter activity) and GO:0015250 (water channel activity) from the “molecular function” class.

The day-peaking genes (656) grouped in nine clusters had expression maxima at or before midday and were mostly of a moderate (4–16) amplitude of expression, except for clusters **15** and **16** (expression amplitude up to 64). Numerous among the day-peaking genes were those related to photosynthesis and the chloroplast (Table S2). However, a global analysis found only one over-represented GO category, GO:0031072 (heat shock protein binding) from the “molecular function” GO class (Figure S4).

The most numerous cluster group comprised 2,538 genes peaking at dusk (**24–32**). For clusters **24,25,26,27** (263 genes), the peak of expression was rather narrow, while for the remaining clusters a fairly constant induction of expression was found between roughly midday and midnight (**28,30**) or even for most of the 24-hour period (**29,31,32**). For the latter genes one should rather speak of a fairly short-lasting drop in expression around dawn/morning. The amplitude of expression of the dusk-peaking genes was moderate (4–16, clusters **24,25,27,28,29**) or low (2–4, clusters **26,30,31,32**). The dusk group of genes not only was the largest, but also stood out in the global GO analysis by containing numerous over-represented GO categories from all three GO classes (Figures S5, S6 and S7): GO:0042723 (thiamine-containing compound metabolic process), GO:0006520 (cellular amino acid metabolic process), GO:0006457 (protein folding), and GO:0006412 (translation) from the “biological process” class, GO:0003735 (structural constituent of ribosome), GO:0004576 (oligosaccharide transferase activity), GO:0016741 (transferase activity, transferring one-carbon groups), GO:0004518 (nuclease activity), and GO:0003723 (RNA binding) from “molecular function”. Although in the “cellular component” class only two lowest-rank categories were over-represented, GO:0005840 (ribosome) and GO:0031974 (membrane-enclosed lumen), in fact the entire “cellular component” class (GO:0005575) was over-represented.

### Search for *cis*- and *trans*-regulatory elements

In search for a molecular basis of the concerted gene regulation apparent in the clustering presented above, we performed a bioinformatic analysis of the regulatory regions of the cycling genes and also looked for potentially involved transcription factors. In the first approach, to narrow down the search to likely functional sites we used comparative genomics and aligned maize upstream sequences (500 bp 5′ from the translation start site) with those of orthologous genes from *S. bicolor* to identify conserved regions. Such conserved regions were then searched for transcription factor-binding sequences.

For most of the clusters one or more over- or under-represented transcription factor-binding sequences were identified (Table 1). The clusters grouping the night- and the dawn-peaking genes usually showed over-represented sites, while the dusk-peaking clusters more often showed under-representation of certain transcription factor-binding sequences. The day-peaking genes were unlike the others as they generally showed neither over- nor under-representation of individual transcription factor-binding sequences. The only exception here was cluster **23**, comprising 363 genes of rather low expression amplitude, among which sequences recognized by three Dof-type transcription factors, Dof2, PBF and MNB1A, were enriched. Three transcription factor-binding sequences were enriched in more than one cluster. In clusters **8** and **12** of similar profiles, bZIP911-binding sequences were enriched, in clusters **12** and **25** of profiles opposite to each other AGL3-binding sequences were over-represented, and in clusters **12** and **29**, also of mutually opposite profiles, HMG-IY.

**Table 1:** Over- and under-represented transcription factor-binding sequences in promoters of maize cycling genes.

Cluster group	Cluster	Transcription factor
nighttotal: 5 clusters	**2**	(+)GAMYB**
	**3**	(+)HMG1*
	**5**	(−)Agamous**
dawntotal: 9 clusters	**7**	(+)Athb1*
	**8**	(+)bZIP911*, (+)bZIP910***
	**9**	(−)Agamous*
	**11**	(+)Myb.ph3*
	**12**	(+)AGL3*, (+)HMG-IY**, (+)bZIP911*
daytotal: 9 clusters	**23**	(+)PBF, (+)MNB1A, (+)Dof2*
dusktotal: 9 clusters	**25**	(+)AGL3*
	**27**	(−)Athb1*
	**28**	(+)Dof3*
	**29**	(+)HMG-IY*
	**30**	(−)GAMYB*, (−)Agamous*, (−)bZIP910*
	**31**	(+)Agamous**
	**32**	(−)HMG1**, (−)PBF*, (−)MNB1A*, (−)Dof2*

Evolutionarily conserved maize gene upstream regions shared between *Z. mays* and *S. bicolor* were searched for specific sequences with the ConSite program. Results are given for individual gene clusters from Figures 1, 2, 3 and 4. Listed are only clusters for which a transcription factor-binding sequence was found under- or over-represented.

Abbreviations: (−) under-representation, (+) over-representation;

*p-value<0.05,

**p-value<0.01,

***p-value<0.001.

The second approach was a search for the *bona fide* clock-associated regulatory sequences identified in *A. thaliana*: EE (evening element), AAAATATCT [4], and CBS (CCA1 binding site), AA(A/C)AATCT [30]. In this case we searched putative promoter regions (500 bp upstream of the translation start codon) of the cycling maize genes. We found the EE and CBS sequences in, respectively, 0.68% and 8.68% of all maize genes (270 and 3,444 genes out of 39,656). Among the 4,898 cycling maize genes under analysis, 84 (1.73%) contained the EE sequence and 247 (5.08%) the CBS sequence. The EE sequence was present mostly in the dusk-peaking genes, while CBS was distributed evenly among all gene clusters. No statistical analysis could be performed for individual gene clusters or even the four cluster groups because of the low numbers of the CBS and EE sequences found.

**Figure 5:**
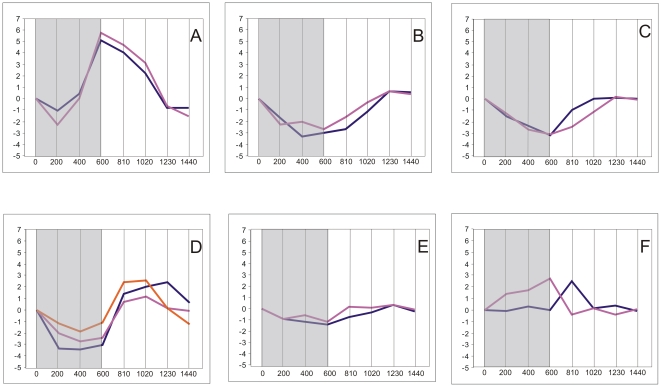
Diel expression profiles of genes potentially involved in biological clock in maize leaves. Genes are defined according to Ariadne Pathway Studio description (see Material and Methods), with *Z. mays* annotations (Zm) when available or *A. thaliana* ones (At). A. LHY (LATE ELONGATED HYPOCOTYL), blue line: At: LHY (average for two probes: MZ00001133 and MZ00020536); purple line: At: LOC100281091 (average for two probes: MZ00014272 and MZ00036336). B. TOC1 (TIMING OF CAB1), blue line: At: TOC1 (probe: MZ00032446); purple line: At: Os11g0157600 (average for two probes: MZ00019763 and MZ00021845). C. GI (GIGANTEA); blue line: Zm: gigz1a (average for two probes: MZ00013980 and MZ00013976); purple line: Zm: gigz1b (average for four probes: MZ00036517, MZ00017821, MZ00013979, MZ00026616). D. Pseudo-response regulators: blue line: At: APRR3 (probe MZ00026527); purple line: At: APRR7 (probe: MZ00015603); orange line: Zm: LOC100285170 (probe: MZ00029712). E. blue line: At: LKP2 (LOV KELCH PROTEIN 2, probe: MZ00057436); purple line: Zm: ELF4 (EARLY flowering 4 protein, probe: MZ00022121). F. blue line: Zm: CHS (chalcone synthase, average for two probes: MZ00044081 and MZ00040585); purple line: Zm: ZGT (circadian clock coupling factor, LOC100285216, probe: MZ00019982).

The third approach was a search for known factors potentially involved in the diel regulation of maize leaf gene expression. To this end, we manually inspected the gene characteristics assigned by the Ariadne Pathway Studio. Several of the maize cycling genes (Figure 5) turned out to be components of plant biological clocks [32]. They included homologs of genes encoding LHY protein, the TOC1 (Timing of CAB) transcription factor, GIGANTEA protein, pseudo-response regulators PRR3, PRR5 and PRR95, all showing high amplitude of diurnal expression (Figure 5A–D), as well as LKP2 (LOV KELCH PROTEIN 2) and ELF4 (EARLY flowering 4 protein) of very low amplitude of diurnal expression (barely meeting the criteria of “cycling”, Figure 5E). LHY, TOC1 and GIGANTEA had two gene variants each; the expression profiles within each paralog pair were similar, but shifted in their day-part relative to each other by one time point (see Figures 5A–C for details).

**Figure 6:**
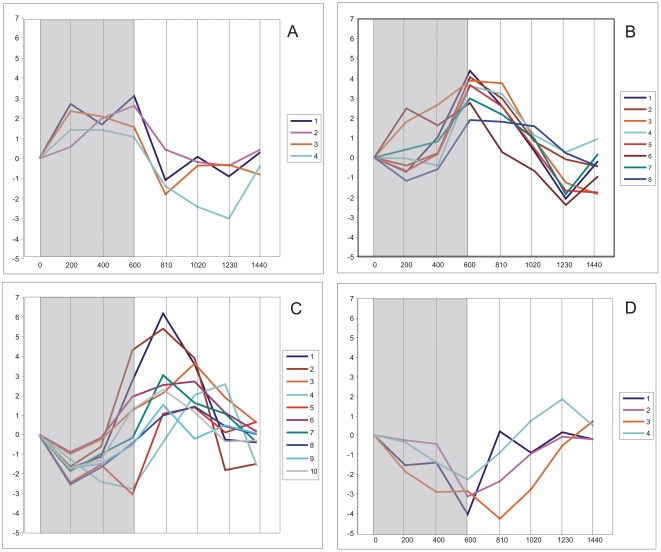
Transcription factors of high amplitude of expression peaking at different times of diurnal cycle. Transcription factors are defined according to Ariadne Pathway Studio description (see Material and Methods), with *Z. mays* annotations (Zm) when available or *A. thaliana* ones (At). A. Transcription factors peaking at night: 1. Zm: R2R3MYB-domain protein, probe: MZ00003796; 2. At: basic helix-loop-helix (bHLH) family protein, probe: MZ00020771. 3. At: myb family transcription factor, probe: MZ00050532. 4. At: COL9 (CONSTANS-LIKE 9), average for two probes: MZ00019724 and MZ00022266. B. transcription factors peaking at dawn: 1. At: myb family transcription factor, probe: MZ00005509. 2. At: AT-HSFA7A; DNA binding/transcription factor, probe: MZ00036996. 3. At: DNA-binding family protein, probe: MZ00023293. 4. zag2 (Zea AGAMOUS homolog2), probe: MZ00026250. 5. At: transcription factor, probe: MZ00032037 6. Zm: R2R3MYB-domain protein, probe: MZ00032119. 7. At: AT-HSFB4, DNA binding/transcription factor, probe: MZ00017901. 8. At: zinc finger (B-box type) family protein, probe: MZ00018166. C. transcription factors peaking at day: 1. At: regulator of chromosome condensation (RCC1) family protein, probe: MZ00024739. 2. At: zinc finger (B-box type) family protein, probe: MZ00055422. 3. Zm: SANT/MYB protein, probe: MZ00028951. 4. At: BZO2H3 (basic leucine zipper O2 homolog 3); DNA binding/transcription factor, probe: MZ00028419: 5. At: myb family transcription factor, probe: MZ00013329. 6. Zm: SANT/MYB protein, probe: MZ00051801. 7. At: PAP2 (PHYTOCHROME-ASSOCIATED PROTEIN 2); transcription factor, probe: MZ00033555: 8. At: CDF1 (CYCLING DOF FACTOR 1), probe: MZ00022960. 9. Zm: HYH (transcription factor HY5), probe: MZ000004193. 10. At: CDF2 (CYCLING DOF FACTOR 2); DNA binding/protein binding/transcription factor, probes: MZ00006024, MZ00020826. D. Transcription factors peaking at dusk: 1. Zm: NAC1 (NAC1 transcription factor), probe: MZ00023973. 2. At: AT-HSFA6B; DNA binding/transcription factor, probe: MZ00001331. 3. At: transcription factor jumonji (jmjC) domain-containing protein, probe: MZ00030647. 4. Zm: Histone H3.2, probe: MZ00023377.

Apart from the above components of the TOC1/LHY biological clock, elements of some other biological clocks were found among the cycling maize genes, such as CHS (chalcone synthase) and ZGT (circadian clock coupling factor ZGT), but the rhythmicality of their expression was only poorly marked (Figure 5F). In addition to the elements of plant biological clocks, numerous other transcription factors (Figure 6) showed strong rhythmicality and amplitude of expression comparable to those of some components of the *Arabidopsis* circadian clock. The dawn- and day-peaking gene cluster groups were particularly rich in transcription factors showing strong amplitudes of expression.

## Discussion

More than one tenth of the *ca.* 40,000 maize microarray probes analyzed showed statistically significant changes in expression of their corresponding genes in the third juvenile leaf (the first fully autotrophic one [20]) in a 24-h cycle. This is a much smaller fraction than that of genes undergoing diurnal changes of expression in *A. thaliana* found by Bläsing *et al.* [10] with the Affymetrix microarray platform (between 30 and 50%), but comparable to the one reported by Schaffer *et al.* [11] with the use of their own microarrays (11%). In a recent paper [18], Hayes *et al.* reported *ca.* 23% of expressed genes to cycle diurnally in the adult maize leaf. Since in their experiment only *ca.* 42% of the *ca.* 105,000 probes printed on the Agilent microarrays used were expressed, the overall fraction of the rhythmically expressed genes relative to the whole set at the microarray (*ca.* 9.5%) is similar to that reported here. In another recent paper by Khan *et al.* [19], *ca.* 10% of *ca.* 13,000 transcripts examined showed diurnal rhythms. One should bear in mind, however, that evaluation of microarray data strongly depends on the experimental design, quality of hybridization, statistical parameters (*e.g.*, when for our data an FDR correction of 0.05 was used instead of 0.01, the number of transcripts showing significant changes in expression increased almost two-fold), and the number of biological replications. Therefore, a quantitative comparison of different microarray experiments is extremely difficult.

In our work, most of the maize transcripts with a rhythmic diel pattern of expression show a relatively low amplitude of expression, *i.e.* below four-fold. There are, however, hundreds of genes of high (4- to 16-fold) and tens of genes of very high (above 32-fold) amplitude of expression. In adult maize leaves [18] the median amplitude of expression (defined by the authors as peak/trough ratio) was reported to be *ca.* 5, that is slightly higher than found here for juvenile leaves. They also observed many genes with expression amplitudes exceeding 20.

One should note that our gene-by-gene analysis of the gene expression in juvenile maize leaves (suppl. materials) did not show any particular response of genes involved in carbohydrate metabolism. The only exception was the over-represented GO category GO:0005975 (carbohydrate metabolic process) among the dawn-peaking gene group (Figures S2, S3). A detailed analysis of genes from this over-represented group shows that most of them are genes related to cell wall formation or lipid metabolism, and only few to starch metabolism or glycolysis. This is in contrast to the results of [10] who reported that in *A. thaliana* genes assigned to sucrose and starch metabolism were particularly abundant among those found to cycle diurnally and showed a high expression amplitude. These differences may reflect the divergent diurnal regulation of carbon partitioning in C3 and C4 plants as postulated by [33] which, in those authors' opinion, could have important implications for diurnal growth pattern and metabolism in these plants. Unlike dicots, maize exports a substantial portion of carbohydrates from leaves during the day [34] and is a moderate starch accumulator [35]. Additionally, maize sucrose phosphate synthase (SPS of class I, [35]), the key enzyme determining carbon distribution between sucrose and starch, is different from that in many other plants. Altogether, it seems that sucrose and starch metabolism in the maize leaf could be regulated differently than in *A. thaliana*, explaining the observed differences in the diurnal regulation of expression of genes related to carbohydrate metabolism in these two species.

When the results of our experiments were being analyzed, two papers addressing diel patterns of gene expression in maize were published [18], [19]. This prompted us to compare our data with those reported. One should note that the three studies in question differed fundamentally in all aspects of experimental design: plant material (adult leaf or ear, V14–15 stage/whole shoot of young seedling, V1 stage/juvenile leaf, V3 stage), growth conditions (natural photo- and thermoperiod in the field/constant light and temperature during material collection, germination and growth under controlled photoperiod and constant temperature/controlled photo- and thermoperiod), microarrays (Agilent with *ca.* 100,000 probes/Affymetrix with *ca.* 13,000 probes/Maize Oligonucleotide Array Project with *ca.* 40,000 probes), and the criteria used to identify rhythmically expressed genes. Two columns in Table S1 show which of the transcripts identified in our study were also found to cycle by the two earlier papers. The data of Khan *et al.* [19] could be analyzed directly, since they supplied GRMZM identifiers (from the Maize Genome Sequencing Project) for all their genes. We found that *ca.* 400 of genes from our report were also defined as showing diurnal rhythmicality by Khan *et al.* [19], which corresponds to *ca.* 30% of their set. Among those genes were many related to transport, in particular aquaporins. Unfortunately, the vast majority of genes from the study by Hayes *et al.* [18] were not listed and thus could not be analyzed here. For this reason we only could consider those few that were listed. Because of this limitation only 56 transcripts could be identified as common between that study and ours; these transcripts were chiefly related to the circadian clock components and the light phase of photosynthesis. Common to all three studies were several transcripts related to the universal components of the circadian oscillator, the light phase of photosynthesis, and five more: thi1-1 (thiamine biosynthesis 1), VTE5 (vitamin E pathway gene5), ATPO2 (polyamine oxidase2), F3H9.20 (undefined), and umc2762 (LOC100193329, ortholog of zinc finger (B-box type) family protein) (Table S1).

Barring the study of Hayes *et al.* [18] that could not be analyzed fully, the above comparative analysis shows that for a substantial fraction of genes defined as diurnally-regulated the classification does not depend on the technical setup of experiments, the algorithms used, or even the physiological age of the plant studied.

### Clustering

To distinguish physiologically relevant responses we resorted to objective clustering of the temporal profiles of gene expression. The method used for the clustering allows the number of the output clusters to be optimized, producing a manageably low number of the most differentiated and fairly homogeneous clusters [28]. When analyzing the data shown in Figures 1, 2, 3 and 4 one notices that some clusters have in fact very similar profiles to each other, differing mostly in the amplitude of expression. Therefore, such “related” clusters were further assembled into groups for subsequent functional analyses. While the clustering of genes according to their expression profiles was done automatically basing on objective criteria, our construction of the cluster groups was done manually and, as such, may be found controversial. Nevertheless, such grouping was necessary, since most of the individual clusters were too small to allow meaningful Gene Ontology statistical analyses. Besides, it also greatly facilitated classification and discussion of the results in terms of plant physiology. We also attempted objective (algorithmic) clustering of all the cycling genes according to their expression peak alone, but the resulting huge clusters were visibly heterogeneous, therefore this approach was not developed further. Based on the general shape of the expression profiles during the 24-h period we distinguished four cluster groups: night, dawn, day, and dusk (see Results). Since the genes within the above groups showed similar expression profiles, some common physiological roles could be expected for them.

The most common were genes with expression maxima towards and during the dark phase: 51% of the cycling genes peaked at dusk and 13% around midnight. This is another apparent difference with *Arabidopsis* since in the latter species the highest portion of genes peaked at dawn under a driven diurnal photoperiod and thermoperiod [10] similar as in our experiments. Rather unexpectedly, our data are also substantially different in this aspect from those on the adult maize leaf reported by Hayes *et al.* [18], who grouped their cycling genes into six bins according to expression peak. In the juvenile leaf, dusk-peaking genes comprised over half of all the cycling ones, while in the adult leaf the “12 h” bin, roughly corresponding to our “dusk” group, included only *ca.* 23% of the cycling genes. The second most numerous group in the juvenile leaf was that of dawn-peaking genes (21%), corresponding to 15% in the adult leaf. In contrast, in the adult leaf the genes peaking during the day (four and eight hours after sunrise) were highly abundant (*ca.* 25 and 8%, respectively), while in the juvenile leaf they were the least abundant (*ca.* 14%). Also the genes peaking at night were substantially more abundant in the adult leaf (*ca.* 26%) than in the juvenile one (12%). A direct comparison of our results with those of [18] must be done cautiously due to the numerous setup differences between the two experiments, including, among others, field *versus* growth chamber conditions, microarray platform, slightly different timing of sample collection, and also different data analysis approaches. Nevertheless, these striking dissimilarities in the abundance of genes peaking at particular phases of the 24-h period are unlikely to be due to the technical differences alone. We believe that they rather reflect the most prominent biological difference between the two experiments, namely the physiological stage of the plants studied: we used juvenile seedlings at the V3 stage, while Hayes *et al.* [18] investigated adult plants at the V14–15 stage. In one-week old maize seedlings the highest fraction of genes were those peaking at the subjective dusk, as in our experiment [19].

### GO analysis

We applied Gene Ontology analysis of the diel transcriptome changes to follow the plant activity at the molecular level throughout the 24-h period (Figures S1, S2, S3, S4, S5, S6 and S7). Most of such activity is stimulated just before and at night: according to the over-represented GO categories, the dark period involves massive induction of genes related to “gene expression”, “translation”, “cellular amino acid metabolic process”, “protein folding”, and “protein modification process”, *i.e.* the most important processes for any living organism. Dawn is a time for induction of expression of genes involved in “protein modification process” (different genes than those induced at night) as well as transport processes including water channels and transporters of ammonia and its methylated derivatives. During the day, no particular activity of maize was found at the molecular level, except for a group of genes encoding proteins related to “heat shock protein binding”. A detailed analysis based on the functional gene annotations showed that, as could be expected, many photosynthesis-related and plastid genes were expressed during the day, however, no over-representation of this gene group was found. In general, our data show that for maize seedlings the most critical time, when profound gene expression changes take place, is between evening and dawn. Also in the adult leaf an important over-represented group highly expressed at “late afternoon” (similar to our “evening” designation) were genes related to RNA processing and modification [18].

### Promoter analysis

The comparative genomics search conducted here highlighted a high proportion (70%) of putative promoter regions of maize genes containing sequences conserved between maize and its close relative C4 grass *S. bicolor* even though transcription factor-binding sequences were only rarely found there by the ConSite software. Such conservation indicates likely regulatory regions of those genes.

When the conserved putative regulatory regions were analyzed for each gene cluster individually, one or more over- or under-represented transcription factor-binding sequences were identified for most of the clusters (Table 1), indicating possible common mechanisms of transcription regulation of the genes from a given cluster.

Among the several transcription factors whose binding sites were found to be enriched in a gene cluster, only one, Dof2, had earlier been shown to function in maize leaves. Dof2 is a repressor of Dof1 that targets the promoter of a C4-type phosphoenolpyruvate (PEP) carboxylase [36]. Interestingly, the Dof2-binding sequence was enriched among genes from cluster **23** that peak around midday (Figure 3), when induction rather than repression of expression of PEP carboxylase might be expected. One should also note that cluster **23** comprises no genes related to photosynthetic carbon metabolism (Table S1). The functions of the other transcription factors from Table 1 are unknown or are related to processes taking place in organs other than leaf. According to the literature, Dof protein PBF activated γ-zein promoter in developing maize seeds [37], bZIP910 and bZIP911 transcripts were found in *Antirrhinum* flowers, but not in green parts of the plant [38], and Myb.ph3 protein was found by means of immunocytolocalization in petal epidermis of *Petunia* [39]; none of them has been reported to be related to circadian rhythms. However, some of the transcription factors whose binding sequences were found to be enriched have been reported to be related to developmental processes in plants. Agamous, AGL3 and Athb1 are involved in flowering in *A. thaliana* [40], and GAMYB is involved in processes regulated by gibberellins and abscisic acid induced in the aleurone layer as well as in floral initiation, stem elongation, anther development and seed development [41]. Since numerous plant developmental processes are interrelated with the circadian clock, a role of the above transcription factors in diurnal rhythmicality could be expected.

One should notice that the set of transcription factor-binding sequences in the ConSite database is rather limited. Further progress in experimental determination of transcription factor-binding sites in grass genomes should allow identification of other transcription factors involved in the diel regulation of gene expression in maize.

We extended the search for transcription factor-binding sequences in the cycling maize genes to the evening element and CBS sequences that had earlier been identified in *Arabidopsis* as statistically enriched in upstream regions of genes regulated by the TOC1/LHY oscillator [4], [30]. In contrast to *Arabidopsis*, relatively few such sequences were found in the upstream regions of the cycling maize genes: only 1.73% of them contained the EE sequence and 5.08% the CBS sequence. In the whole maize genome, the EE and CBS sequences are present in, respectively, 0.68% and 8.68% of genes. The corresponding figures for *A. thaliana* are 1.15% and 14.14% (387 and 4,750 genes out of 33,602), respectively (our calculations). Thus, the abundance of the evening element is similar in the two genomes, but the CBS sequence is significantly (p<0.05) more abundant in the *Arabidopsis* genome compared with the maize one. These numbers yet again underscore the differences between the genomes and regulatory mechanisms of the model plant *Arabidopsis* and maize.

### Specificity of biological clocks in maize

Although it was not the primary aim of this paper to characterize biological clocks in maize, some conclusions could be made concerning the endogenous diurnal regulation of gene expression. A detailed analysis of genes showing diel changes in expression identified maize orthologs of known components of *Arabidopsis* circadian oscillator, TOC1, LHY, GIGANTEA, PRR3, PRR5, and PRR95, as well as LKP2 and ELF4 [32]. The overall behavior of those clock components seems to be similar in both species, although the amplitude of expression was rather low for the maize LKP2 and ELF4 transcripts (Figure 5E). Virtually identical genes were found to cycle diurnally, with highly similar patterns, in the shoot of one-week old seedlings under free-running experiment conditions [19] and in the adult leaf under field conditions [18]. Conservation of circadian clock components was also found in rice and poplar under both free-running and driven diurnal photoperiod and thermoperiod [17]. In addition to the genes reported here, those authors found several other biological clock components.

Unlike in *Arabidopsis* or the adult maize leaf, the presence of two TOC1/LHY oscillators as well as two GIGANTEA variants can be postulated in juvenile maize leaves with slightly different time profiles of expression. Within each pair both paralogs reach the maximum expression at the same time, but the two curves are shifted relative to each other by one time point (210 min) on their descending (LHY) or ascending (TOC1, GIGANTEA) arm. This slightly off-set pattern could reflect different anatomical localization of the two postulated clocks. Spatial separation of tissue-specific plant circadian clocks has already been suggested in [6].

Since maize is a C4 plant, with the photosynthetic apparatus distributed between Kranz mesophyll (KMS) cells and bundle sheath (BS) cells, these two tissues could be the potential locations where separate circadian oscillators could function. Their slightly different profiles would reflect the differences in the photosynthetic apparatus in KMS and BS cells, *e.g.*, a lack of PSII in BS, uneven distribution of many enzymes, and the dedication of KMS cells to sucrose synthesis and the BS cells to starch production [42]. This hypothesis is contrary to the results of a study by Sawers *et al.* [43] on gene expression profiles in KMS and BS of the maize leaf with the use of microarrays from the Maize Oligonucleotide Array Project, University of Arizona, Tucson, as in our study. That study did not report a different distribution of appropriate mRNAs in the KMS and BS cells (our search of their data). One should note, however, that they were using an earlier, two-slide version of the microarrays and a specific statistical model, which makes problematic a direct comparison of the results of these two projects. Strict verification of our conjecture regarding the tissue localization of expression of the clock genes is only possible by a direct approach using qRT PCR in samples of KMS and BS cells or *in situ* hybridization.

In addition to the discussed duality of the LHY/TOC1 oscillator, some other interesting features of the maize biological clock could be deduced from our results. This concerns the diel regulation of chalcone synthase (CHS) and the circadian clock coupling factor ZGT (Figure 5F). In *Arabidopsis*, CHS has been found to exhibit rhythmic expression with a peak at dawn [6]. In contrast, our results only show a transient induction of expression of CHS 210 min after dawn (Figure 5F) instead of the rhythmic changes found in *Arabidopsis*. In turn, the circadian clock coupling factor ZGT has been shown to follow a circadian rhythm in tobacco, with almost no expression during the night and a peak 8 h after dawn. Conversely, the maize ortholog LOC100285216 was well expressed throughout the night with a substantial fall just after dawn (Figure 5F). These two cases demonstrate that the general conclusions regarding plant biological clocks based on results of model plant studies need not always apply to other plant species. Instead, direct studies on economically important crops are necessary for detailed characterization of the biological clocks operating in these plants, bearing in mind the pivotal role of biological rhythms in the practical aspects of plant physiology, such as the timing of germination, flowering, fruit/seed development, dormancy, *etc.* This conclusion is supported by our observation that tens of transcription factors demonstrate rhythmic expression in maize, including COL9, CDF1, CDF2, HYH and PAP2 (Figure 6) involved in photoperiodic regulation, as well as many others whose functions have not been related with rhythmicality until now. Interestingly, four (BZO2H3, CDF2 and orthologs of COL9 and regulator of chromosome condensation (RCC1) family protein) of 25 transcription factors with the highest amplitude of expression (Figure 6) were also found to cycle in the young shoot [19]. Additionally, one transcription factor (a zinc finger B-box type family protein) was found to cycle diurnally in the young shoot [19], adult leaf [18] and first autotrophic leaf (this report). These transcription factors are strong candidate components of a biological clock(s) in maize.

### Conclusions

Microarray data deliver information on plant functioning only at the transcriptome level, so their relevance to the true effector level of physiology must not be taken for granted. The value of such data is additionally limited by the often sketchy description of genes represented by the microarray probes. Nevertheless, the powerful statistical and bioinformatic tools designed for global analyses of gene expression allow one at least to formulate scientific hypotheses for further verification concerning the molecular mechanisms governing the functioning of an organism. Bearing in mind the above concerns, some general conclusions can be drawn from our results. The first one concerns the mainly nocturnal life of the plant. This largely neglected aspect of plant functioning should be studied more thoroughly. Plants, by their nature, depend on light, so the most attention has been paid (also for practical reasons) to processes occurring during the day. It seems, however, that the nocturnal phase of plants' life is actually very active and thus deserves more attention. The second general conclusion is that although some universal mechanisms might be responsible for the diel regulation of gene expression in plants, here represented by orthologs of the genes encoding components of *Arabidopsis* circadian oscillators, other factors and systems determining details of the diurnal regulation of gene expression seem to differ between *A. thaliana* and maize. In general, monocots, comprising numerous valuable crops of which maize is a representative, should not be assumed to function following what is known for *A. thaliana*, but rather ought to be studied directly.

## Supporting Information

Figure S1 Gene Ontology categories from “biological process” class significantly over-represented among transcripts in cluster group “night”. Only the relevant fragment of GO graph is shown; over-represented categories are highlighted. Numbers attributed to a GO term indicate, respectively: total number of transcripts described by this GO term in the population/size of the population; number of transcripts described by this GO term among cycling transcripts/total number of cycling transcripts.
(TIF)

Figure S2 Gene Ontology categories significantly over-represented among transcripts in cluster group “dawn”, GO class: “biological process”. Only the relevant fragment of GO graph is shown; over-represented categories are highlighted. Numbers attributed to a GO term indicate, respectively: total number of transcripts described by this GO term in the population/size of the population; number of transcripts described by this GO term among cycling transcripts/total number of cycling transcripts.
(TIF)

Figure S3 Gene Ontology categories significantly over-represented among transcripts in cluster group “dawn”, GO class: “molecular function”. Only the relevant fragment of GO graph is shown; over-represented categories are highlighted. Numbers attributed to a GO term indicate, respectively: total number of transcripts described by this GO term in the population/size of the population; number of transcripts described by this GO term among cycling transcripts/total number of cycling transcripts.
(TIF)

Figure S4 Gene Ontology category from “molecular function” class significantly over-represented among transcripts in cluster group “day”. Only the relevant fragment of GO graph is shown; over-represented category is highlighted. Numbers attributed to a GO term indicate, respectively: total number of transcripts described by this GO term in the population/size of the population; number of transcripts described by this GO term among cycling transcripts/total number of cycling transcripts.
(TIF)

Figure S5 Gene Ontology categories significantly over-represented among transcripts in cluster group “dusk”, GO class: “biological process”. Only the relevant fragment of GO graph is shown; over-represented categories are highlighted. Numbers attributed to a GO term indicate, respectively: total number of transcripts described by this GO term in the population/size of the population; number of transcripts described by this GO term among cycling transcripts/total number of cycling transcripts.
(TIF)

Figure S6 Gene Ontology categories significantly over-represented among transcripts in cluster group “dusk”, GO class: “molecular function”. Only the relevant fragment of GO graph is shown; over-represented categories are highlighted. Numbers attributed to a GO term indicate, respectively: total number of transcripts described by this GO term in the population/size of the population; number of transcripts described by this GO term among cycling transcripts/total number of cycling transcripts.
(TIF)

Figure S7 Gene Ontology categories significantly over-represented among transcripts in cluster group “dusk”, GO class: “cellular component”. Only the relevant fragment of GO graph is shown; over-represented categories are highlighted. Numbers attributed to a GO term indicate, respectively: total number of transcripts described by this GO term in the population/size of the population; number of transcripts described by this GO term among cycling transcripts/total number of cycling transcripts.
(TIF)

Table S1 Annotation of cycling genes and clustering of gene expression profiles. Clusters created with pvclust were further combined manually into four groups (“night”, “dawn”, “day” and “dusk”).
(XLS)

Table S2 Annotation of genes in enriched Gene Ontology categories in cluster groups “dusk”, “dawn” and “day”. For “night” group only one GO category was enriched so it is not presented here. Capital letters in square brackets correspond to GO categories: F = molecular function, C = cellular component, P = biological process.
(XLS)
