# Supplementary figures and images for: Rhythmic Diel Pattern of Gene Expression in Juvenile Maize Leaf
Source: PLoS One. 2011 Aug 17;6(8):e23628. doi: 10.1371/journal.pone.0023628 (PMC3157397; doi:10.1371/journal.pone.0023628)

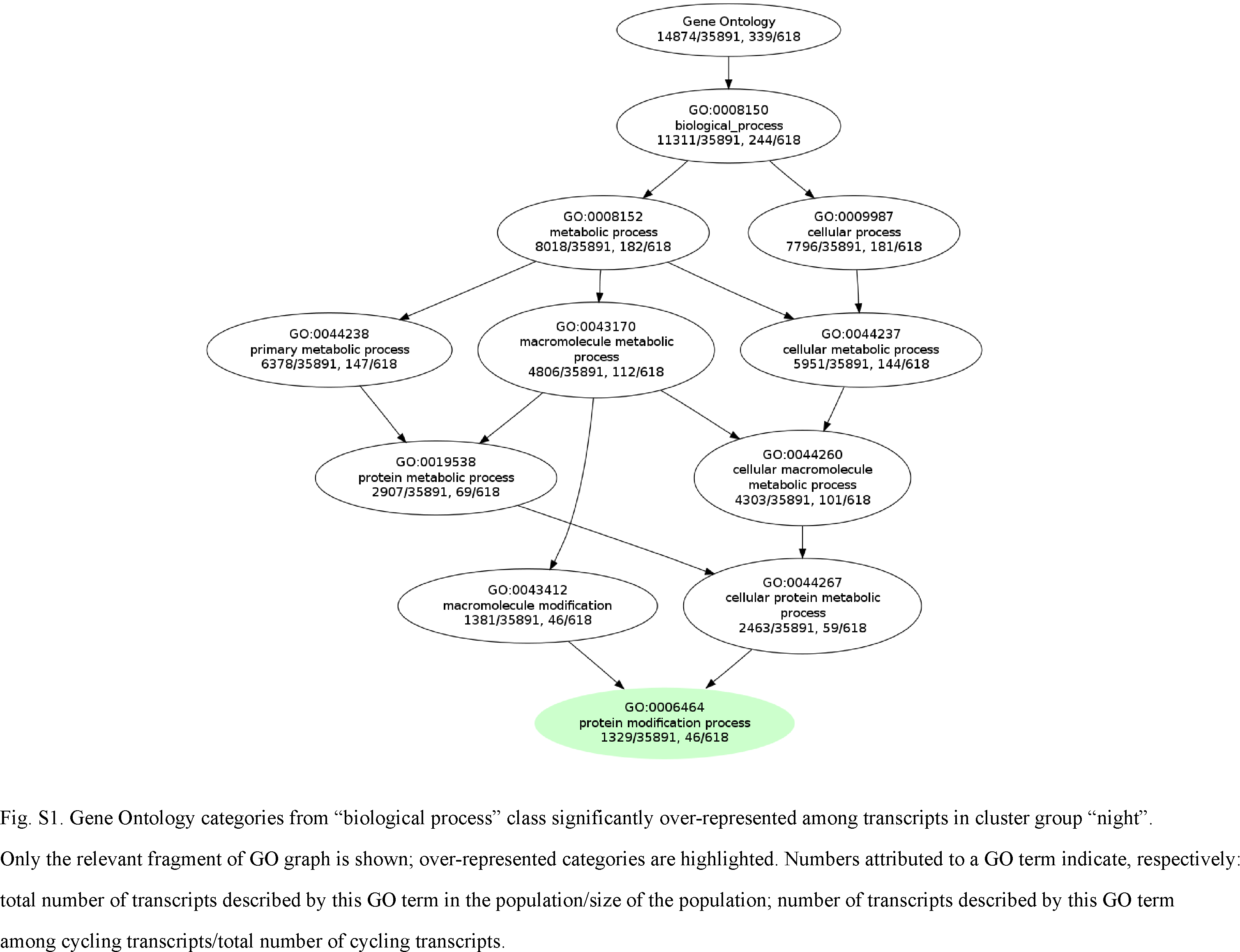

Supplement: Figure S1 — Gene Ontology categories from “biological process” class significantly over-represented among transcripts in cluster group “night”. Only the relevant fragment of GO graph is shown; over-represented categories are highlighted. Numbers attributed to a GO term indicate, respectively: total number of transcripts described by this GO term in the population/size of the population; number of transcripts described by this GO term among cycling transcripts/total number of cycling transcripts. (TIF) [file pone.0023628.s001.tif]

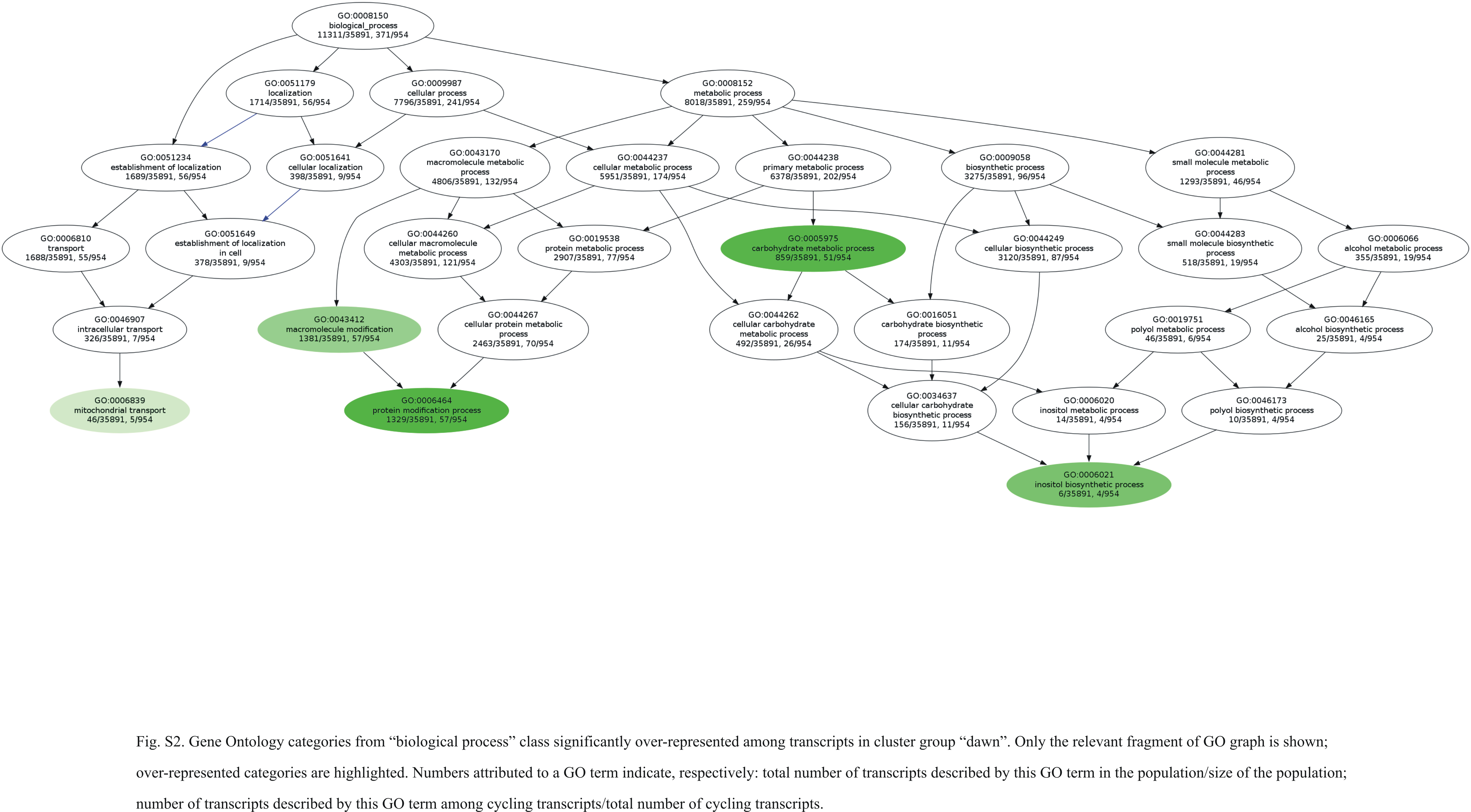

Supplement: Figure S2 — Gene Ontology categories significantly over-represented among transcripts in cluster group “dawn”, GO class: “biological process”. Only the relevant fragment of GO graph is shown; over-represented categories are highlighted. Numbers attributed to a GO term indicate, respectively: total number of transcripts described by this GO term in the population/size of the population; number of transcripts described by this GO term among cycling transcripts/total number of cycling transcripts. (TIF) [file pone.0023628.s002.tif]

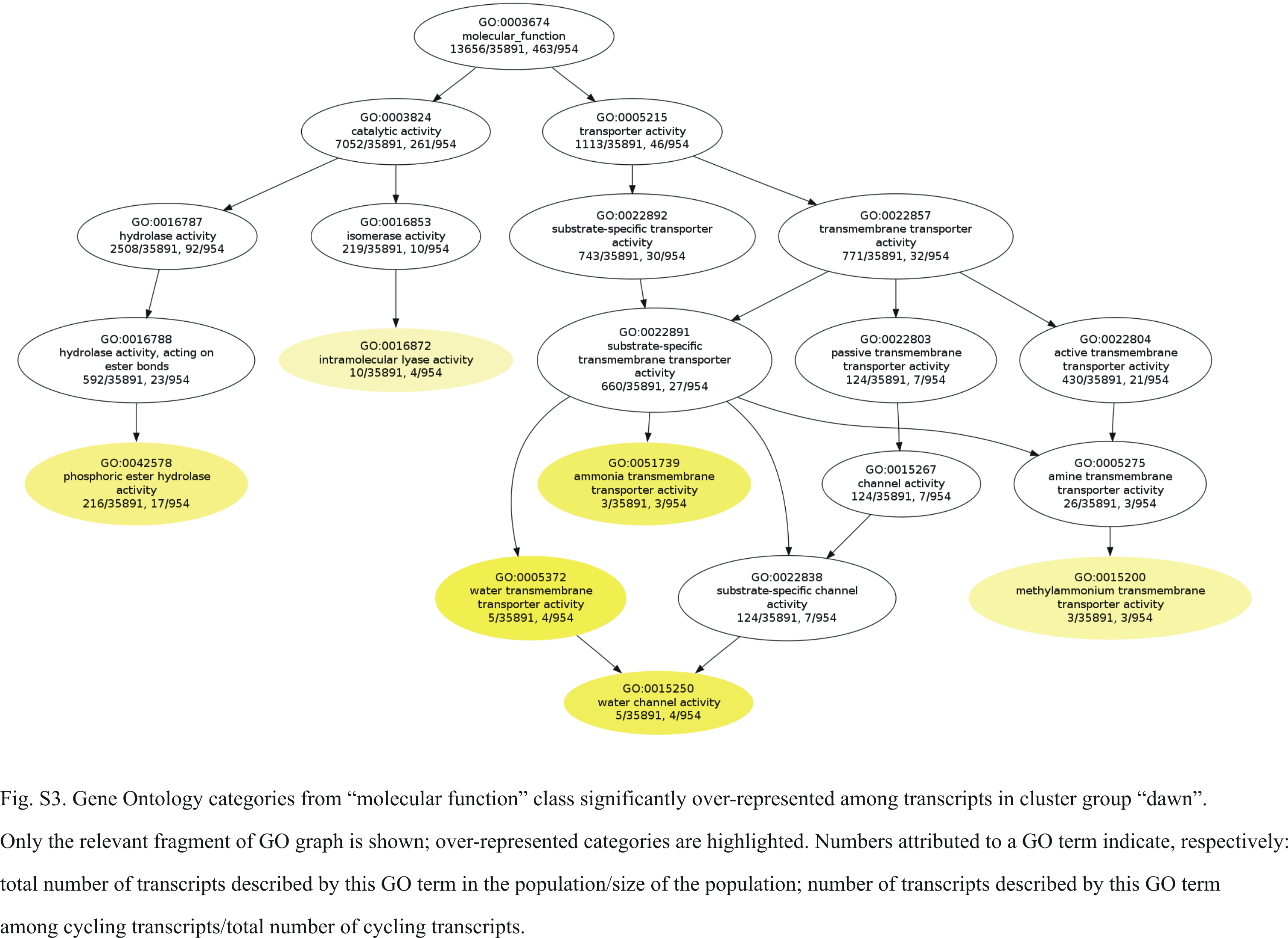

Supplement: Figure S3 — Gene Ontology categories significantly over-represented among transcripts in cluster group “dawn”, GO class: “molecular function”. Only the relevant fragment of GO graph is shown; over-represented categories are highlighted. Numbers attributed to a GO term indicate, respectively: total number of transcripts described by this GO term in the population/size of the population; number of transcripts described by this GO term among cycling transcripts/total number of cycling transcripts. (TIF) [file pone.0023628.s003.tif]

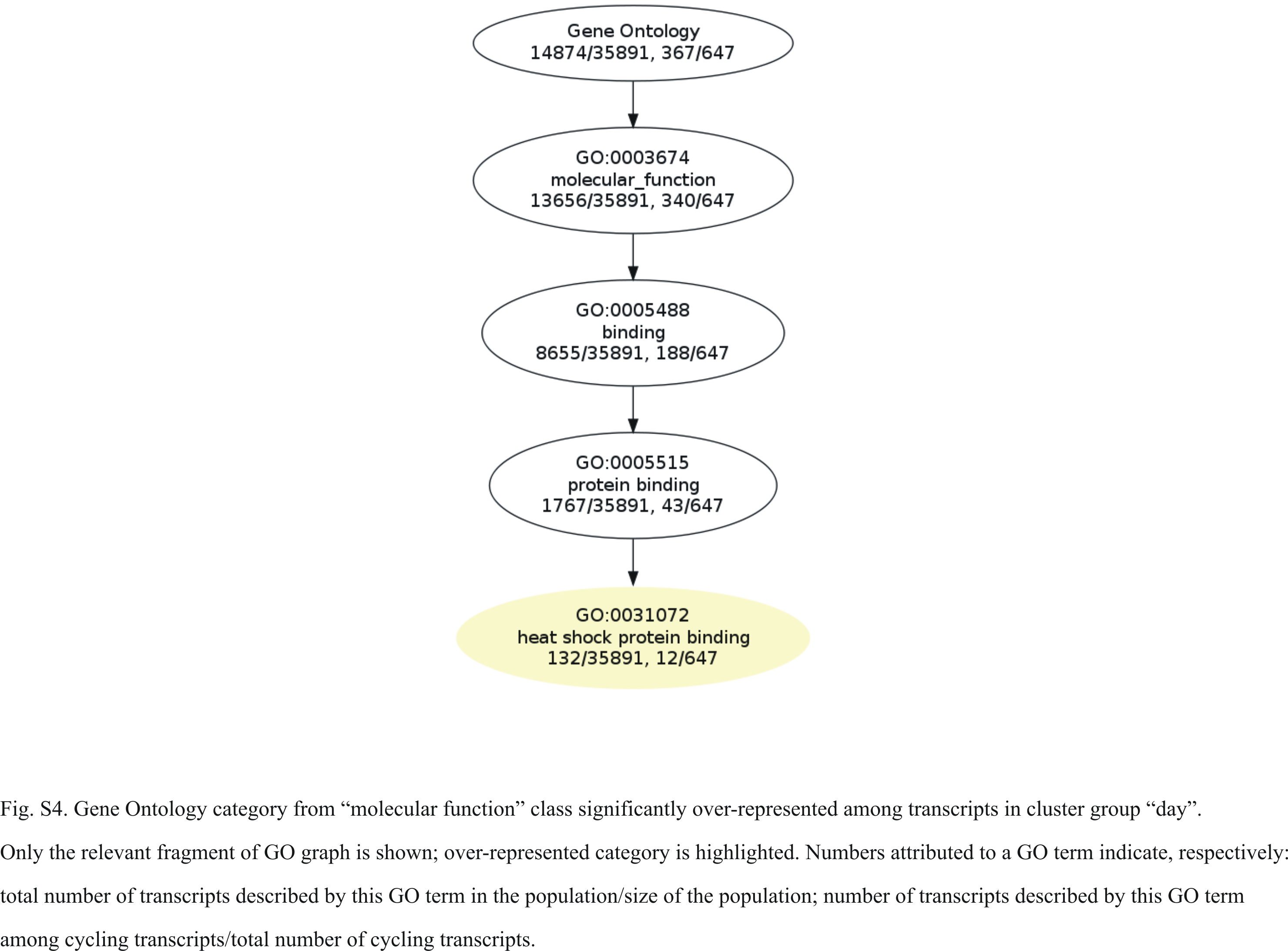

Supplement: Figure S4 — Gene Ontology category from “molecular function” class significantly over-represented among transcripts in cluster group “day”. Only the relevant fragment of GO graph is shown; over-represented category is highlighted. Numbers attributed to a GO term indicate, respectively: total number of transcripts described by this GO term in the population/size of the population; number of transcripts described by this GO term among cycling transcripts/total number of cycling transcripts. (TIF) [file pone.0023628.s004.tif]

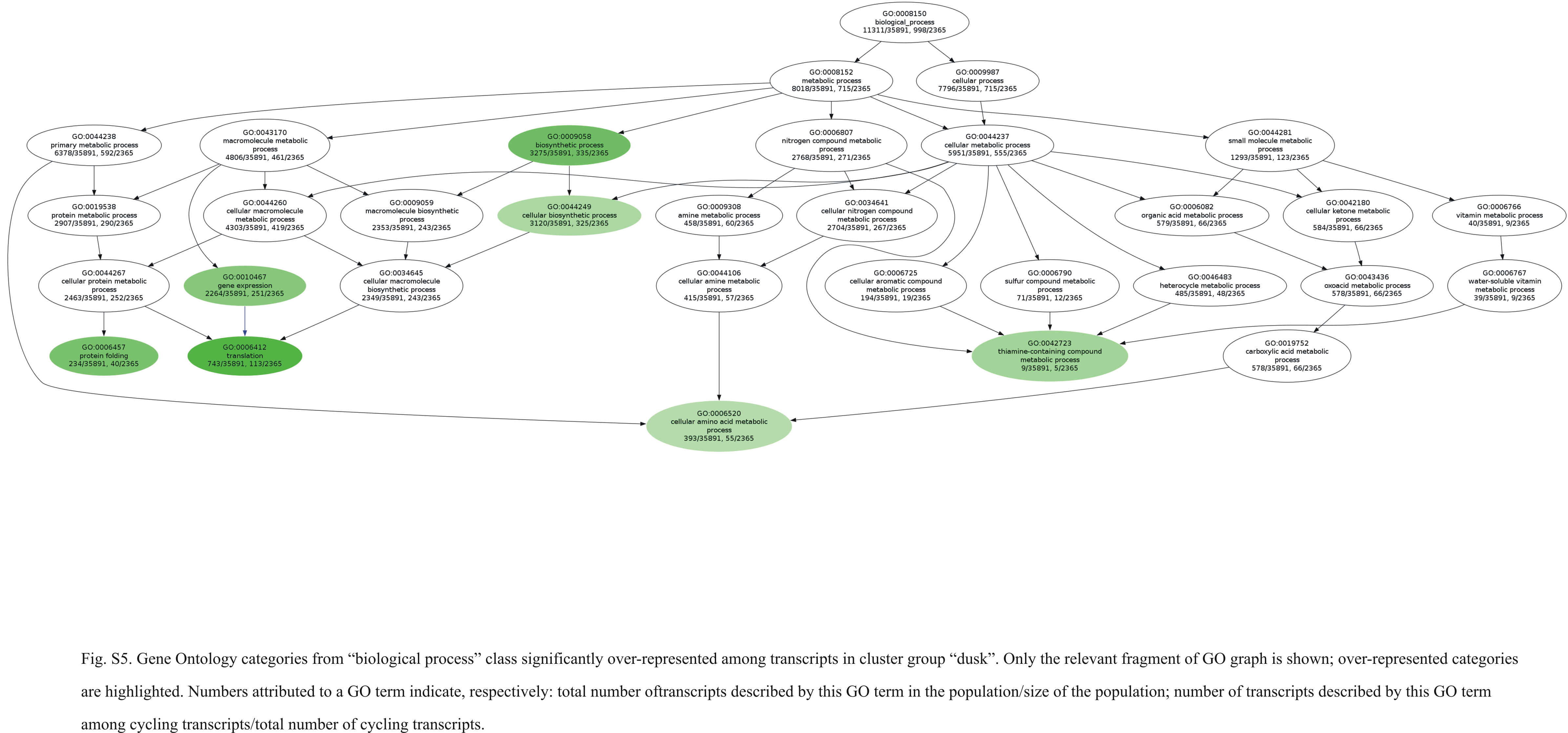

Supplement: Figure S5 — Gene Ontology categories significantly over-represented among transcripts in cluster group “dusk”, GO class: “biological process”. Only the relevant fragment of GO graph is shown; over-represented categories are highlighted. Numbers attributed to a GO term indicate, respectively: total number of transcripts described by this GO term in the population/size of the population; number of transcripts described by this GO term among cycling transcripts/total number of cycling transcripts. (TIF) [file pone.0023628.s005.tif]

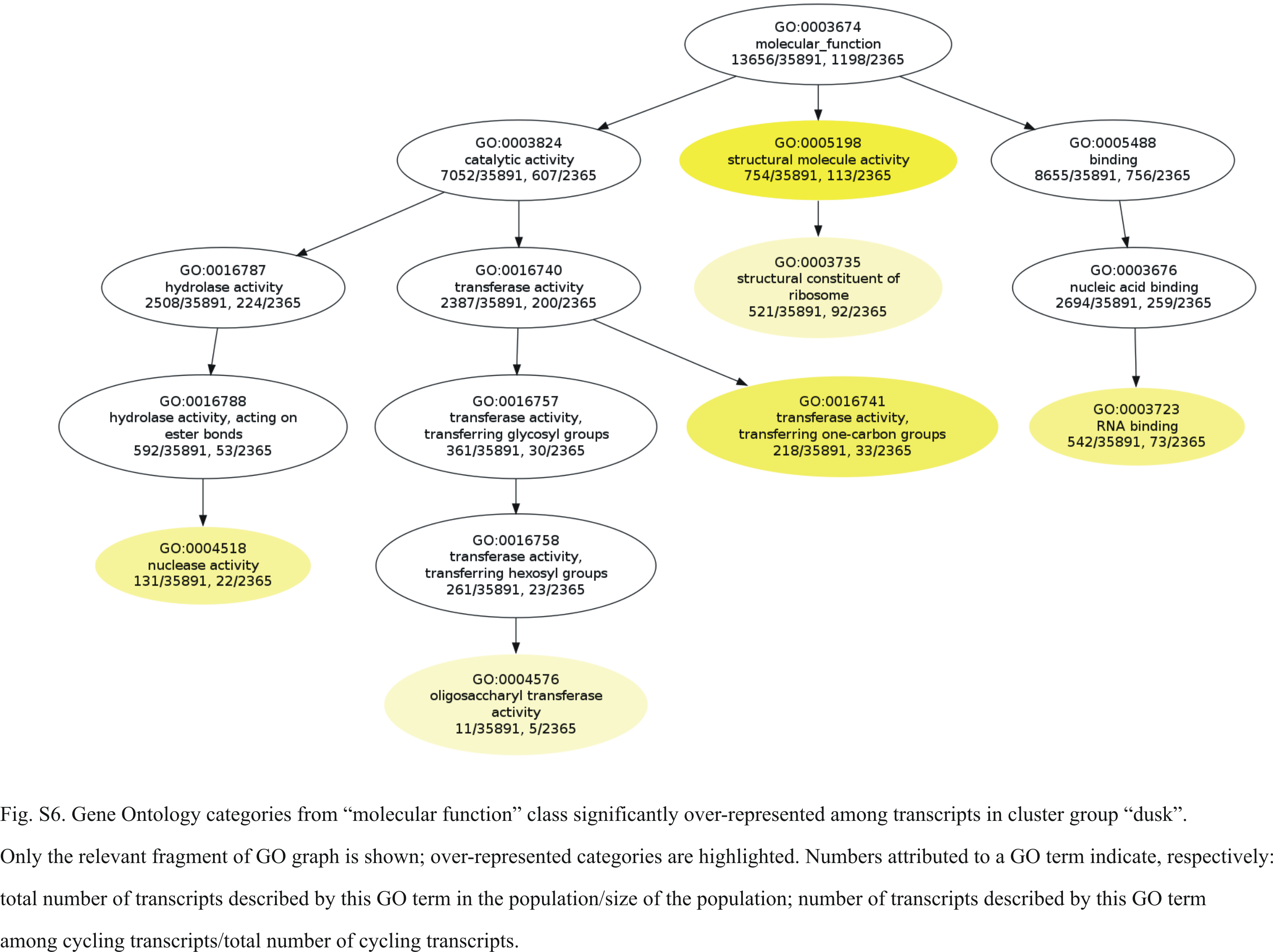

Supplement: Figure S6 — Gene Ontology categories significantly over-represented among transcripts in cluster group “dusk”, GO class: “molecular function”. Only the relevant fragment of GO graph is shown; over-represented categories are highlighted. Numbers attributed to a GO term indicate, respectively: total number of transcripts described by this GO term in the population/size of the population; number of transcripts described by this GO term among cycling transcripts/total number of cycling transcripts. (TIF) [file pone.0023628.s006.tif]

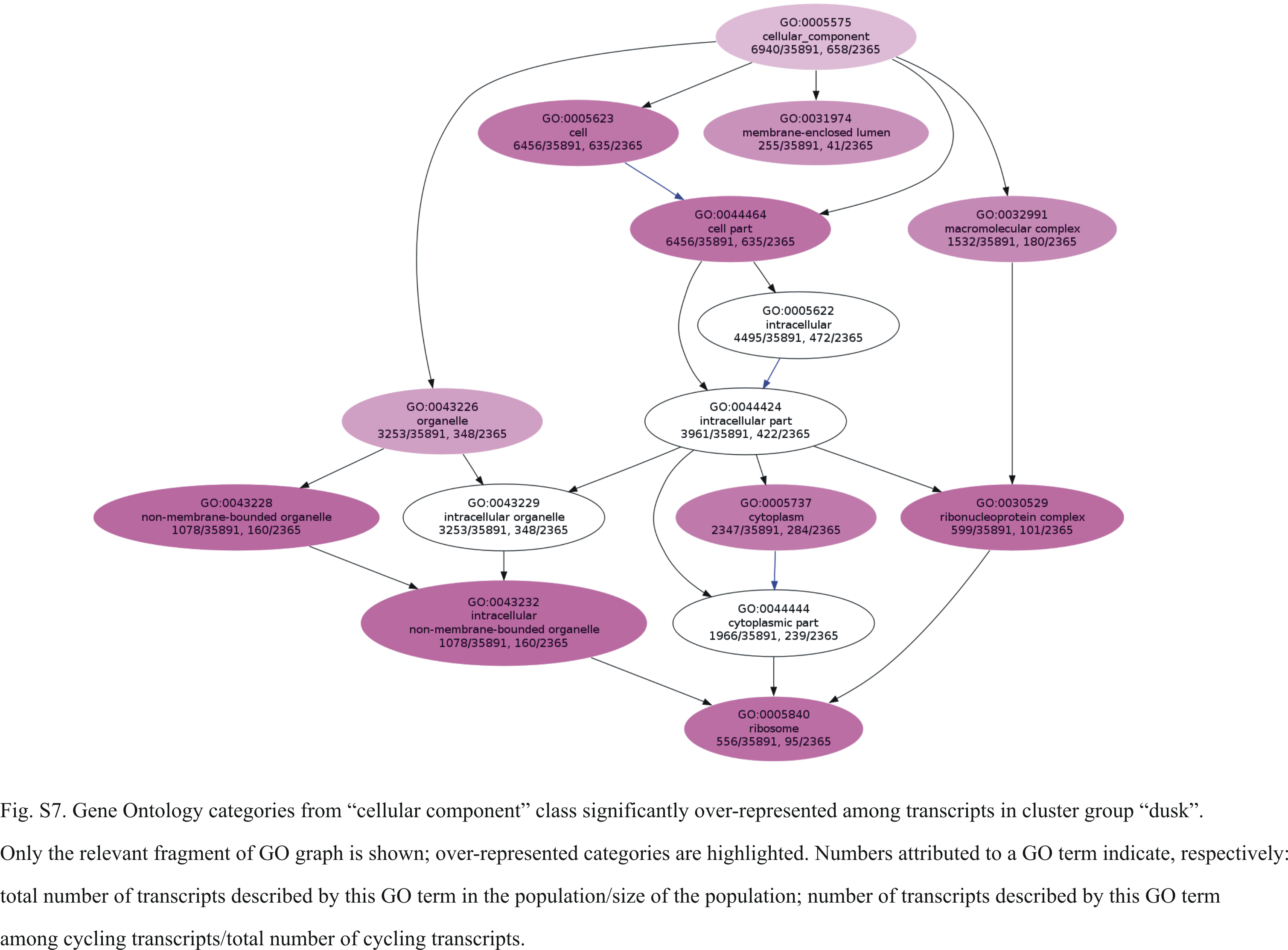

Supplement: Figure S7 — Gene Ontology categories significantly over-represented among transcripts in cluster group “dusk”, GO class: “cellular component”. Only the relevant fragment of GO graph is shown; over-represented categories are highlighted. Numbers attributed to a GO term indicate, respectively: total number of transcripts described by this GO term in the population/size of the population; number of transcripts described by this GO term among cycling transcripts/total number of cycling transcripts. (TIF) [file pone.0023628.s007.tif]
